# Developing Climate Change and Health Impact Monitoring with eHealth at the South East Asia Community Observatory and Health and Demographic Surveillance Site, Malaysia (CHIMES)

**DOI:** 10.3389/fpubh.2023.1153149

**Published:** 2023-12-01

**Authors:** Sandra Barteit, David Colmar, Syahrul Nellis, Min Thu, Jessica Watterson, Darwin Gouwanda, Till Bärnighausen, Tin Tin Su

**Affiliations:** ^1^Heidelberg Institute of Global Health (HIGH), Faculty of Medicine and University Hospital, Heidelberg University, Heidelberg, Germany; ^2^South East Asia Community Observatory (SEACO) and Global Public Health, Jeffrey Cheah School of Medicine and Health Sciences, Monash University Malaysia, Bandar Sunway, Malaysia; ^3^Jeffrey Cheah School of Medicine and Health Sciences, Monash University Malaysia, Bandar Sunway, Malaysia; ^4^School of Engineering (Mechanical), Monash University Malaysia, Bandar Sunway, Malaysia; ^5^Department of Global Health and Population, Harvard T.H. Chan School of Public Health, Boston, United States; ^6^Harvard Center for Population and Development Studies, Harvard T.H. Chan School of Public Health, Cambridge, MA, United States

**Keywords:** climate change, fitness trackers, wearables, weather stations, 3D-print, global health, Malaysia

## Abstract

**Background:**

Malaysia is projected to experience an increase in heat, rainfall, rainfall variability, dry spells, thunderstorms, and high winds due to climate change. This may lead to a rise in heat-related mortality, reduced nutritional security, and potential migration due to uninhabitable land. Currently, there is limited data regarding the health implications of climate change on the Malaysian populace, which hinders informed decision-making and interventions.

**Objective:**

This study aims to assess the feasibility and reliability of using sensor-based devices to enhance climate change and health research within the SEACO health and demographic surveillance site (HDSS) in Malaysia. We will particularly focus on the effects of climate-sensitive diseases, emphasizing lung conditions like chronic obstructive pulmonary disease (COPD) and asthma.

**Methods:**

In our mixed-methods approach, 120 participants (>18 years) from the SEACO HDSS in Segamat, Malaysia, will be engaged over three cycles, each lasting 3 weeks. Participants will use wearables to monitor heart rate, activity, and sleep. Indoor sensors will measure temperature in indoor living spaces, while 3D-printed weather stations will track indoor temperature and humidity. In each cycle, a minimum of 10 participants at high risk for COPD or asthma will be identified. Through interviews and questionnaires, we will evaluate the devices’ reliability, the prevalence of climate-sensitive lung diseases, and their correlation with environmental factors, like heat and humidity.

**Results:**

We anticipate that the sensor-based measurements will offer a comprehensive understanding of the interplay between climate-sensitive diseases and weather variables. The data is expected to reveal correlations between health impacts and weather exposures like heat. Participant feedback will offer perspectives on the usability and feasibility of these digital tools.

**Conclusion:**

Our study within the SEACO HDSS in Malaysia will evaluate the potential of sensor-based digital technologies in monitoring the interplay between climate change and health, particularly for climate-sensitive diseases like COPD and asthma. The data generated will likely provide details on health profiles in relation to weather exposures. Feedback will indicate the acceptability of these tools for broader health surveillance. As climate change continues to impact global health, evaluating the potential of such digital technologies is crucial to understand its potential to inform policy and intervention strategies in vulnerable regions.

## 1 Introduction

### 1.1 Malaysia and climate change-induced exposures

In recent decades, extreme weather events have become increasingly frequent and severe in Southeast Asia (1, 2). According to the fourth Intergovernmental Panel on Climate Change (IPCC) assessment report (3), the mean surface air temperature in Southeast Asia has been rising for several decades, with a 0.1–0.3°C increase in each decade between 1951 and 2000 (4). This includes more heat waves (hot days and nights and fewer cold days and nights), an increase in severe rainstorms, and an increase in tropical cyclones. In 2018, more than 12,000 Malaysians were displaced by floods in four states (Johor, Terengganu, Pahang, and Sabah); more recently, in 2021, over 50 people died in a flood in Selangor with nearly four meters of water, and over 400,000 people were evacuated (5, 6). In Malaysia, heat-related mortality is anticipated to increase, along with an increase in vector-borne diseases (7). Additionally, other forecasts indicate that under a 400–800 ppm level of CO_2_, rice yield could reduce by 4.6 to 6.1% for every 1°C increase in temperature (8). As agriculture is dependent on weather conditions, climate change is a significant concern (5). Furthermore, coastal erosion and the loss of mangrove trees are directly attributable to the high waves caused by a rise in sea level, which are exacerbated by warmer sea temperatures that may impact fish livelihoods by causing more fish-threatening algal blooms and coral reef bleaching, thereby endangering small-scale as well as food security (9).

In Malaysia, the health paradigm has transitioned from infectious to non-communicable diseases, including asthma, diabetes (10, 11), and other cardiometabolic and respiratory conditions (12). Elevated temperatures have been consistently linked to adverse pulmonary outcomes (13, 14), yet their impact on climate-sensitive populations in Malaysia remains underexplored. D’Amato et al. highlighted that increasing temperatures and associated heat stress can induce or intensify respiratory disease manifestations (15). Notably, heat-exacerbated respiratory conditions such as asthma and COPD appear to be prevalent in Malaysia (16).

This transition is further complicated by the diverse challenges posed by climate change. At the same time, Malaysia is faced with an ageing population, particularly in rural regions. Older individuals often exhibit reduced heat resilience, especially when they have pre-existing conditions such as cardiovascular disorders (17, 18). Furthermore, age-related cognitive decline or impairment may reduce behavioral adaptive capacity (19). Specifically, rural Malaysian areas, characterized by lower socioeconomic status, substandard housing, and limited access to cooling devices like air conditioning, are particularly vulnerable (20, 21). Rural subdistricts in Malaysia exhibit a distinct demographic profile compared to the national average, largely due to prevalent rural-to-urban youth migration (22).

Despite a global uptick in political and research efforts (23), studies on climate change adaptation and mitigation remain limited in Malaysia (5).

Empirical research on the health implications of climate change, as well as adaptation strategies and interventions for marginalized and rural communities, remains limited (5, 24). Adaptation to climate change is vital for climate-vulnerable populations in emerging economies to cope with rising average temperatures and extreme heat events caused by climate change.

### 1.2 South East Asia Community Observatory, Malaysia, as an ideal infrastructure for climate change and health research

Health and Demographic Surveillance Systems (HDSSs) are operational in more than 56 health cohorts throughout Africa and Asia, with some systems tracking population dynamics—including births, deaths, and migrations—for over six decades (25, 26). The INDEPTH network streamlines HDSS implementation in LMICs. HDSS data has enabled a better understanding of infectious diseases, amongst others, such as malaria, HIV/AIDS, and tuberculosis, and the efficacy of interventions (25). Especially in countries without comprehensive health systems, HDSSs provide crucial health data, capturing demographic and health trends (26).

In 2011, Monash University Malaysia launched the South East Asia Community Observatory (SEACO HDSS) in Segamat, Johor’s northernmost district. Operating within five semi-rural sub-districts—Sungai Segamat, Chaah, Bekok, Gemereh, and Jabi—this surveillance site spans around 1,250 km^2^, encompassing nearly 13,000 households and approximately 40,000 individuals (27). The SEACO HDSS operates successful community engagement strategies including regular community consultation meetings. It has hosted numerous research studies on infectious diseases (dengue) (28), non-communicable diseases (diabetes, hypertension, stroke, multi-morbidity) (29, 30), as well as lifestyle and health behavior research (31, 32) in order to assess the disease burden of the population.

With the Climate Change and Health Impact Monitoring with eHealth system (CHIMES) we will implement a system for the continuous monitoring of health and climate metrics within the SEACO HDSS, Malaysia, adopting modules from the Climate Change and Health Evaluation and Response System (CHEERS) (33). This study centers on three tiers of data collection (individual, household, and community) using digital, sensor-based devices.

Our main objectives are as following:

1. Establish and explore best approaches for sensor-based measurements for climate change and health research in the SEACO HDSS:
a. Establish acceptability of sensor-based devices.
b.Determine the reliability of sensor-based devices.
c. Evaluate long-term technical performance of sensor-based devices.
2. Assess the burden of climate-sensitive diseases in populations of the SEACO HDSS:

a. Establish the feasibility of measuring heat strain indicators: lung disease variables, heart rate, activity, sleep.
b. Determine adequacy and usefulness of lung disease measures as heat strain indicator.
c. Investigate the correlation between weather variables, especially temperature and humidity, and lung disease variables.

## 2 Methods and analysis

### 2.1 Study design

The study will employ a convergent mixed-methods approach (34). Both qualitative and quantitative data will be collected at the same time. Qualitative data will be gathered through semi-structured in-depth interviews (IDIs), providing insights into experiences and perceptions. Quantitative data is sourced from log system data, and Likert-scaled questionnaires, and structured activity diaries, offering measurable and statistical information. After the data collection phase, both sets of data are first analyzed separately. Subsequently, they are merged in the interpretation phase, where results from both data types are synthesized, compared, and contrasted. This process allows us to identify areas of convergence, where qualitative and quantitative findings align, as well as areas of divergence, where they might offer different perspectives or insights.

### 2.2 Study 1: feasibility and acceptability study for sensor-based measurements for climate change and health research (individual-, home-, and community-based sensors)

Employing the locally-hosted SurveyCTO platform (35), we will administer questionnaires to gather insights on participants’ usage and experiences with the Garmin vivosmart 5 wearable device (36), the SwitchBot Meter for indoor temperature and humidity measurements (37), and the 3D-printed weather stations (38). We will collect feedback from a group of SEACO HDSS stakeholders, which includes field supervisors, technical staff, and fieldworkers. In-depth interviews (IDIs) will also be conducted with key personnel from SEACO HDSS, such as the director, selected scientists, community representatives, and fieldworkers, to ensure a holistic understanding and comprehensive feedback. We anticipate data saturation for these stakeholder interviews based on the 10 + 3 criterion (39).

Quantitative data will be collected from routine device usage and their diagnostic logs to evaluate the reliability of these sensor-based tools. This data will be juxtaposed with fieldworker questionnaire responses. We will employ five metrics to evaluate device reliability: (i) failure rate of temperature sensors (how often do they fail to make a measurement?), (ii) replacement rate (how often do the sensors technically fail so they need to be fully replaced?), (iii) maximum battery-energy intervals (how often do the devices battery run out of power, especially under heat and humidity stress?), (iv) maximum data collection intervals (how often does data need to be synchronized?), and (v) failure rate of data synchronization (how frequently are data synchronization challenges that result in inaccurate or missing data encountered?).

Data obtained from 3D-printed weather stations will be validated by comparison with data from weather stations in the study area, managed by the Meteorological Service of Malaysia (40), focusing on temperature, humidity and rainfall.

### 2.3 Study 2: burden of climate-sensitive diseases in climate-vulnerable semi-urban and rural populations in the SEACO HDSS

Within our mixed-methods approach, we will conduct an observational panel study to evaluate the feasibility and acceptance of digital spirometry measurements, focusing on lung diseases as potential indicators of heat strain. To identify individuals at an increased risk of lung disease symptoms, specifically chronic obstructive pulmonary disease (COPD) and asthma, we will utilize validated questionnaires, administered by fieldworkers using offline tablets, for initial population screening. Lung function assessments will be conducted in participants’ homes using hand-held, digital spirometers, specifically the Medical International Research (MIR) Spirobank Smart Spirometer. To further understand participants’ self-perceived health burden, we will administer weekly questionnaires comprising Likert-scaled, multiple-choice, and open-ended questions (the complete questionnaire is provided in the Supplementary material).

### 2.4 Study setting

**Figure 1:**
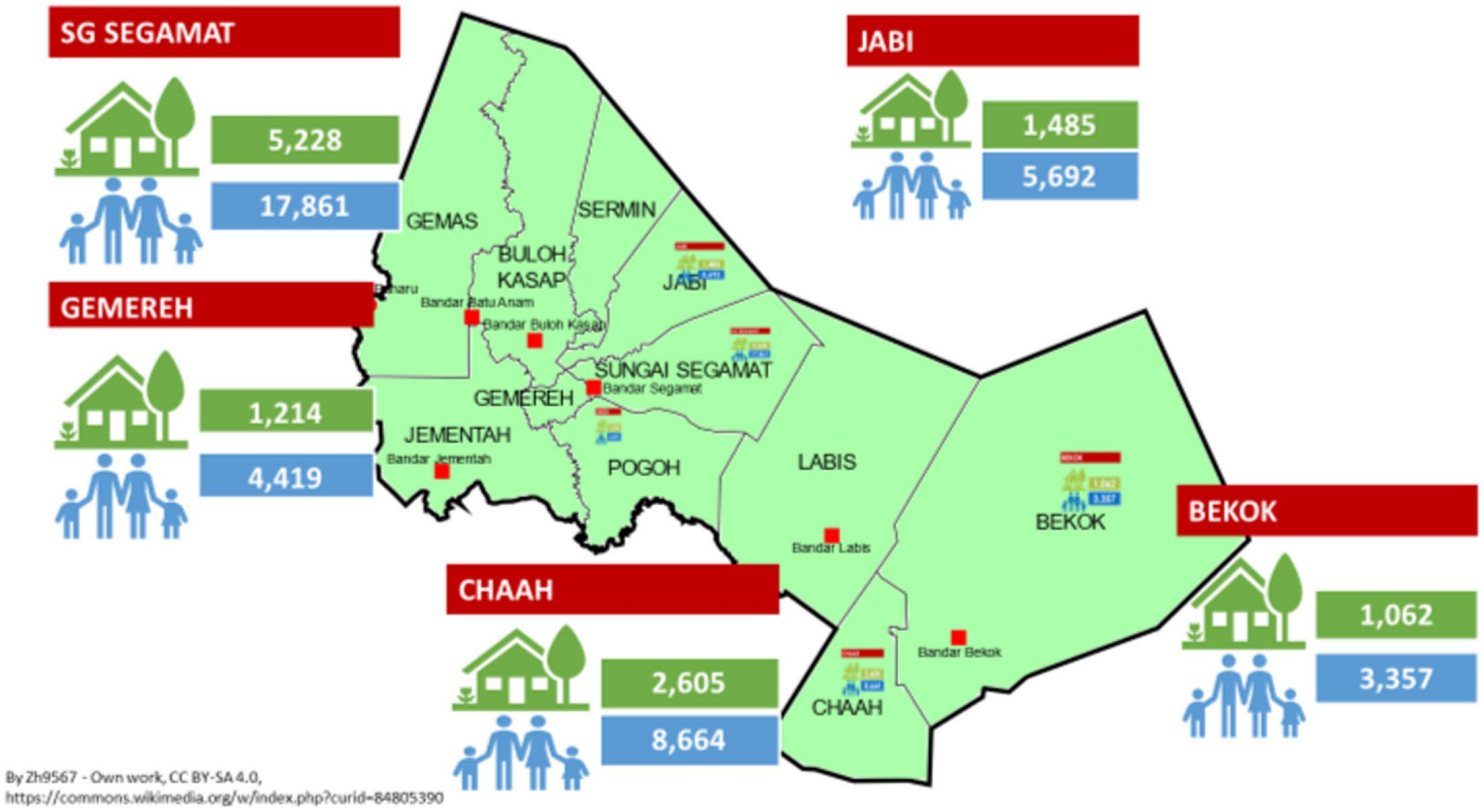
Overview of the SEACO HDSS located in the Segamat region, Malaysia, with its five sub-districts: Sungai (SG) Segamat, Gemereh, Chaah, Bekok, and Jabi.

The SEACO HDSS, located in Segamat, a district in the southern peninsular state of Johor, Malaysia, is managed by Monash University Malaysia and primarily funded by Monash University’s Malaysian and Australian branches. Since 2011, SEACO HDSS has offered longitudinal data on socio-demographics, health, and population (27). The SEACO HDSS encompasses five of 11 sub-districts of Segamat with a total area of roughly 1,250 km^2^ (see Figure 1) and a population of around 40,000 individuals from different cultural and ethnic backgrounds. The demographic profile of SEACO HDSS reflects the ethnic composition of Malaysia: Malay (62.4%), Chinese (17.9%), Indian (9.5%), Orang Asli (2.2%), and other groups (27).

While Malaysia generally experiences light, variable winds, there are four distinct seasons influenced by wind direction: the northeast monsoon, the southwest monsoon, and two inter-monsoon periods (41). The El Niño Southern Oscillation (ENSO) affects rainfall distribution, with El Niño years often being the hottest (42). In 2019, the average temperature was 27.63°C, with peaks at around 33°C and lows at around 24°C (42). Annual precipitation averages 3,085.5 mm, ranging from 200 mm in mid-year to 350 mm towards year-end, with consistent levels year-round (43).

### 2.5 Study participants and recruitment

Eligible participants for this study are individuals aged 18 and above who reside within one of the five SEACO HDSS sub-districts. Our recruitment strategy will reflect the demographic composition of the Segamat District, taking into account age, and gender. The SEACO data manager will spearhead the identification of potential participants. Participation is entirely voluntary, and due to the study’s data collection methods, it will not be anonymous. To ensure a diverse representation, only one member from each household will be selected. Before any study-related activities commence, participants must provide their written informed consent. If at any point a participant decides to withdraw or is lost to follow-up, their participation will be terminated without any repercussions.

Given the possibility of dropouts or refusals, we will adopt an oversampling approach. Our aim is to engage *n* = 100 participants for the three study cycles. However, factoring in an estimated refusal rate of 50%, we anticipate a final sample size of *n* = 120 participants. Each study cycle will be dedicated to a specific sub-district: Jabi, Chaah, or Sungai Segamat.

The study will span nine weeks. While participants might not derive direct benefits from their involvement, the insights gleaned from this research are expected to inform and shape broader initiatives that could benefit the larger community.

### 2.6 Sample size calculation

As the true problem probability is unknown in our study population, we will employ the methodology proposed by Viechtbauer et al. to determine the sample size, using a lower bound for the problem probability (44). Specifically, we aim to be 95% confident in detecting any prevalent problem at 8% (probability 0.08), necessitating a sample size of *n* = 36 participants (refer to the online calculator for pilot study sample sizes (45)). Consequently, our study will comprise three cohorts of *n* = 40 participants each, with every cohort participating for a three-week duration.

### 2.7 Sensor-based devices (individual, home-, and community-based)

Data in this study is stored securely on encrypted, password-protected devices. Initial sensor data is saved on encrypted tablets used by fieldworkers. The SEACO HDSS, ensures encrypted data transfer to the German research team, complying with Monash University Malaysia’s data protection standards. The study timeline, analysis, and results dissemination are detailed in Table 1.

**Table 1:** Timeline of study stages.

Agenda Item	Month											
	1	2	3	4	5	6	7	8	9	10	11	12
Develop protocol												
Obtain ethical clearance												
Publish protocol paper												
Conduct feasibility study Setting up sensor-based measurements (individual, home-based, community-based) Sensor-based data collection												
Data analysis												
Prepare and publish feasibility study manuscript												
Design full study based on feasibility study findings												

SEACO HDSS data resides in PostgreSQL databases, structured according to the HDSS Reference Data Model, which includes individual, social group, and event data. The database administrator, guided by the HDSS steering committee, oversees data access and linkage. SEACO HDSS employs SurveyCTO for data collection and stores data on the ARCD Nectar Research Cloud (46).

#### 2.7.1 Individual sensors

##### 2.7.1.1 Garmin VivoSmart 5 wearable device

The Garmin vivosmart 5 (see Table 2 for details) is a wrist-worn wearable device (worn 24 h per day by study participants). The data is synchronized with the field worker’s tablet through Bluetooth, and the field worker visits the participant’s home every 5–7 days. The wearable device stores up to 14 days of data and has a 7-day battery life. The following variables will be collected:

**Table 2:** Overview of sensor-based devices (individual and home-based sensors) employed as part of this feasibility, acceptability, and reliability study.

	Individual sensor	Individual sensor	Home-based sensor
Image	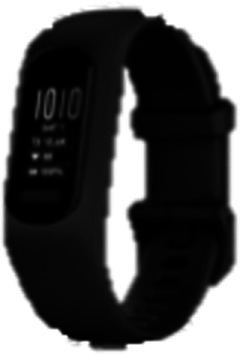	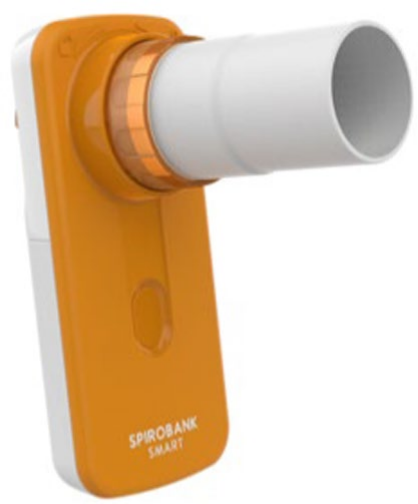	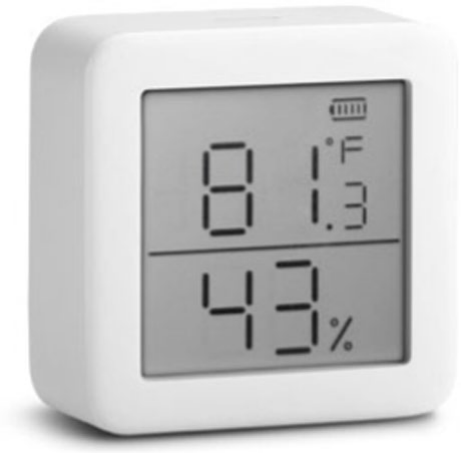
Sensor name and type	Garmin vivosmart 5	MIR Spirobank Smart Oxi Spirometer	SwitchBot Meter
Measurements	Heart Rate (beats per minute) Activity Time (in steps) Sleep Time (hours) Blood Oxygen Saturation (SpO2) Max. volume of oxygen (VO2)	Lung capacity variables: Functional Vital Capacity (FVC) Forced Expiratory Volume in 1 s (FEV_1_) FEV1% (Predicted Forced Expiratory Volume in 1 s) Peak Expiratory Flow Rate Forced Expiratory Flow, average between 25 and 75% of expiration Forced Expiratory Time	Ambient Air Temperature (degrees Celsius) Relative Air Humidity (%)

- daily activity (measured as steps, distance, calories; measured with an accelerometer sensor based on amplitude and periodic pattern)
- heart rate (routinely measured every 10 min with a photoplethysmography sensor; heart rate measured every 1 s only in workout mode (manually enabled) or after 2 min of running)
- sleep (wearable automatically detects sleep and monitors movement during sleep; sleep data include total hours of sleep, sleep stages, sleep movement, and sleep score)

##### 2.7.1.2 Spirobank smart spirometer

The MIR Spirobank Smart spirometer uses a flow sensor (bi-directional digital turbine) to assess a number of lung function parameters in individuals (see Table 2). The tablet-based application (app), which shows the test quality and results of the spirometer test in real-time, receives the measured lung function parameters through Bluetooth. Individual participant data can also be exported in PDF or CSV format. The production company suggests the operating conditions suitable between 5 and 40°C, and humidity of 10–93% Relative Humidity (RH).

#### 2.7.2 Home-based sensor: SwitchBot meter

The SwitchBot Meter is a smart, consumer-grade indoor sensor that measures the ambient air temperature (°C) and relative humidity (see Table 2). The sensor is mounted to the participant’s living room wall or ceiling. The SwitchBot Meter samples data every 4 s, saves up to 5 weeks collected data, and the batteries (removable) are estimated to last around 1 year. The production company specifies the temperature accuracy as +/− 0.7°F, the humidity accuracy as +/− 4% RH, and the working environment as −10 to 60°C and 20–85% RH.

#### 2.7.3 Community-based sensor: 3D-printed weather stations

**Figure 2:**
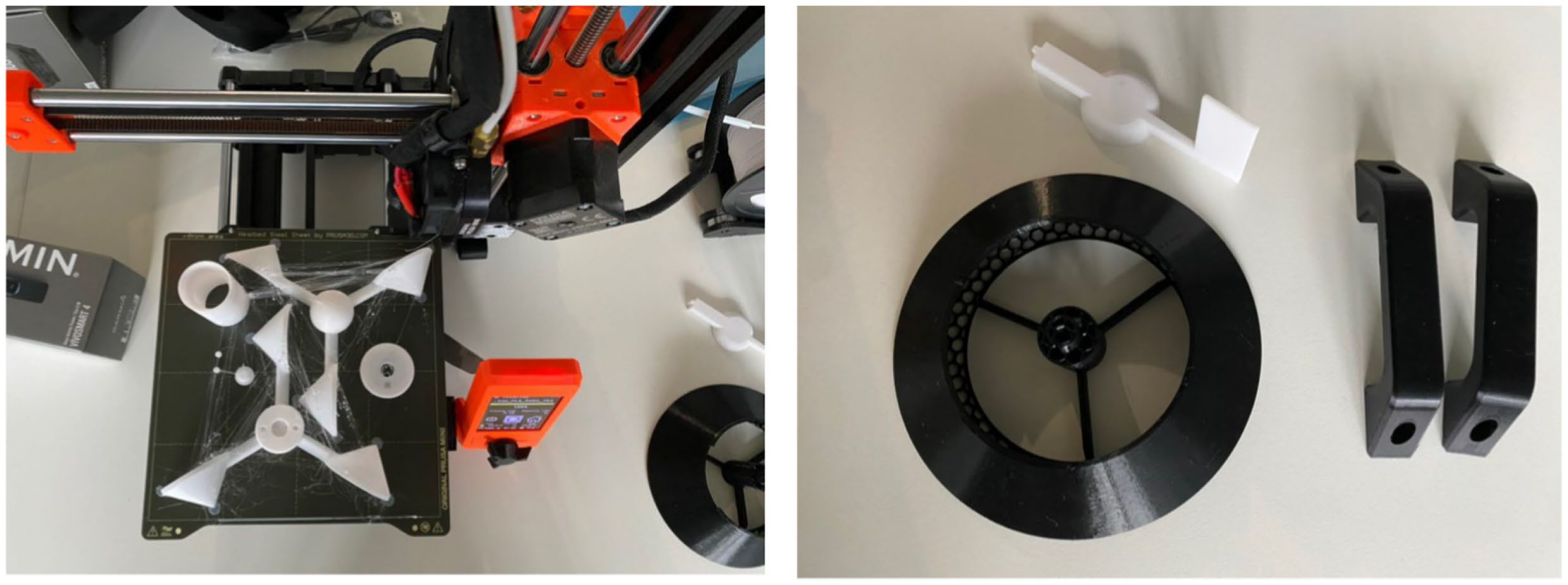
Test 3D-printed parts of the weather station (anemometer, wind vane, weather radiation shield). Image on left hand side shows the 3D-printed parts (in white), on the right-hand side shows the solar radiation shield (printed in black as a test print) on the build platform of the Prusa MINI+ 3D-printer.

Weather stations will be 3D-printed in alignment with the guidelines and validated blueprints provided by the 3D-Printed Automatic Weather Station (3D-PAWS) initiative (38). The designed weather station is equipped to measure parameters such as wind speed, wind direction, rainfall, air temperature, relative humidity, solar radiation, and air pressure. A solar panel, requiring a 5 Volt input, powers the station. Components essential for the construction of these stations are procurable from local hardware vendors in Malaysia. For the 3D-printing process, we will employ the Prusa MINI+ 3D-printer, augmented with a Bond Tec dual extruder to enhance the precision of Acrylic Styrene Acrylonitrile (ASA) printing (refer to Figure 2 for details).

**Figure 3:**
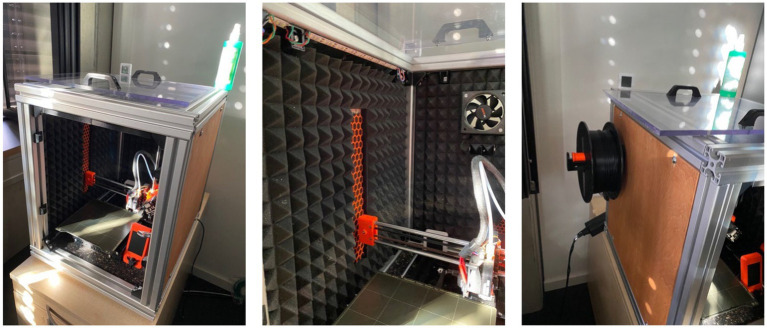
Ventilated, portable enclosure constructed from flame-resistant pressboard for the 3D-printer as seen from the front (left image), the padded view showing the ventilator (middle image), and the provision for the filament spool and power cord (right image).

ASA filament is known for its UV resistance and ability to withstand high temperatures up to 93°C (47). However, it can experience warping due to rapid cooling of filament layers (47). Additionally, ASA emits fumes during printing, necessitating well-ventilated ambient conditions (47). To counter these issues, we use a flame-resistant pressboard enclosure for the 3D-printer (see Figure 3) to maintain stable internal temperatures and reduce fume exposure. Given ASA’s tendency to absorb moisture, a filament dryer is also utilized to prevent moisture-related printing anomalies.

### 2.8 Study proceedings

#### 2.8.1 Evaluating the feasibility, acceptability and validity of individual- and home-based sensors as routine measurements and to measure effects of heat stress

In this study, field staff will receive a 2-day training on using sensor-based devices, administering questionnaires, understanding study goals, and following safety protocols.

Our team will consist of 4–6 fieldworkers collecting data over three weeks in selected sub-study regions: Jabi, Chaah, and Segamat. These regions capture the study population’s diversity. Fieldworkers, selected based on experience, area familiarity, and training, ensure data consistency.

At each cycle’s start, fieldworkers gather participant details, measure height and weight, and administer a pre-screening questionnaire for potential lung diseases, including COPD and asthma [COPD questionnaire: incorporates the population-based screening questionnaire “Could it be COPD?” (48) which was adopted from the Global Initiative for Chronic Obstructive Lung Disease consisting out of five question items; asthma questionnaire: incorporates the symptom-based questionnaire for predicting the presence of asthma questionnaire (Asthma Screening Questionnaire (ASQ) consisting out of six question items (49), refer to Table 3)]. If the lung disease questionnaire outcome shows a high likelihood of study participants’ having a lung disease with a device cutoff ratio of <74% (0.74) forced expiratory volume in 1 s to forced vital capacity ratios [FEV_1_/FVC; ideal cutoff based on a population-health study conducted in Taiwan with the same spirometer device (50)], the field worker conducts a spirometry assessment with those study participants. We measure with the spirometer forced expiratory volume in 1 s to forced vital capacity ratios (FEV_1_/FVC) to identify lung airflow restrictions.

**Table 3:** At the time of baseline data collection, screening questionnaires for chronic obstructive pulmonary disease (COPD) and asthma are administered to determine the likelihood that study participants have COPD or asthma.

Questionnaire: Could it be COPD	
Question item	Response options
Do you cough several times most days?	Yes No
Do you bring up phlegm or mucus most days?	Yes No
Do you get out of breath more easily than others your age?	Yes No
Are you older than 40 years?	Yes No
Are you a current smoker or an ex-smoker?	Yes No
Asthma Screening Questionnaire (ASQ)	
Question item	Response options
Do you cough more than the average person?	Yes No
Do you have a cough that comes mainly from your chest and NOT from your throat?	Yes No
Do you have worsening of the following symptoms when you lie down to sleep?	Yes No
Do you have worsening of the following symptoms after exercise or physical activity?	Yes No
Do you have worsening of the following symptoms after laughing or crying?	Yes No
Do you have worsening of the following symptoms after talking on the phone?	Yes No

Fieldworkers visit weekly to administer questionnaires, gather daily activity data, and check devices.

After the 21-day study, devices are collected, deregistered, cleaned, and data synchronized. Post the nine-week study, in-depth interviews (IDIs) are conducted with fieldworkers, participants, and SEACO HDSS stakeholders to gather feedback on the devices (refer to Supplemenatry material, questionnaire CHIMES).

#### 2.8.2 Feasibility, acceptability, and reliability of community-based, 3D-printed weather stations in the semi-rural context of the SEACO HDSS

Within the SEACO HDSS, we will install two 3D-printed weather stations. Over a nine-week duration, a field worker will conduct weekly visual inspections of the weather station and its immediate environment, subsequently filling out a structured questionnaire to record the station’s condition (refer to Table 4). The data gathered from the 3D-printed weather stations will be cross-referenced with measurements from the closest meteorological station operated by the Malaysian meteorological service.

**Table 4:** Structured questionnaire for evaluating 3D-printed weather stations in the SEACO HDSS.

3D-printed weather station structured questionnaire	
Question item	Response
Date of inspection.	Input of current date.
Location of weather station.	Input of GPS coordinates of weather station location.
Please take pictures of the weather station and surroundings.	Taking pictures with tablet of weather station surroundings.
Have you noticed any visible damages when inspecting the weather station?	Yes No
Please describe in detail the damages of the weather station	Written text typed in tablet.
What have people said about the weather station?	Written text typed in tablet.
Note down any other comments regarding the weather station.	Written text typed in tablet.

### 2.9 Data analysis

Upon completion of data collection, both quantitative and qualitative results will be merged and analyzed to discern any convergence or divergence. Questionnaire data will undergo cleaning, categorization, and thematic analysis. All other statistical analyses will be conducted using the R software. The normality of the data will be assessed using the Shapiro–Wilk test. The Welch-t test will be employed to compare participants based on gender and study arm. Analyses will further categorize participants by gender, socio-economic status, age, and lung status. For error estimation, 95% confidence intervals (CIs) will be reported.

#### 2.9.1 Feasibility of sensor-based devices (individual, household-, and community-based)

##### 2.9.1.1 Reliability

We will monitor reliability of sensor-based devices using five key performance indicators: (i) failure rate, (ii) replacement rate, (iii) maximum battery-energy intervals, (iv) maximum data downloading intervals and (v) failure rate of data synchronization.

The cumulative instances when data was not collected will serve as a measure of reliability. Established metrics, such as the intra-cluster correlation coefficient (ICC) and the Bland–Altman plot (51), will be employed to further evaluate the reliability of the sensor-based devices.

##### 2.9.1.2 Data validity

We will descriptively present data on sleep duration, day- and nighttime heart rate, calorie consumption, activity, spirometer and indoor temperature and humidity. A threshold of ≥20 h of measurements per day will be set for step count and calorie consumption to be considered valid (52). Indoor temperatures and humidity levels will be compared with outdoor temperatures and humidity levels, and we will compute the standard error of the mean (SEM), root mean square error (RMSE), average difference, and the minimum and maximum values.

For the validation of the 3D-printed weather stations, we will obtain data from verified weather stations under the Malaysian Meteorological Department located near our 3D-printed stations. A comparative analysis will be conducted to ascertain the validity and reliability of the measurements from the 3D-printed stations (53). Metrics such as SEM, RMSE, average difference, minimum and maximum values will be computed, and a monthly breakdown of RMSE and correlation coefficients will be provided for the entire deployment duration of the weather station.

##### 2.9.1.3 Data completeness

Completeness of the data will be evaluated based on the consistency of measurements during predetermined intervals (54). To determine the validity of the data from sensor-based devices, specific criteria will be set for each sensor, drawing from prior research and best practices (55, 56). As highlighted by Huhn et al. (57), we will consider the manufacturer-specified measurement interval for non-continuously monitoring sensors, amplified by a factor of 1.5 (57).

##### 2.9.1.4 Data usability

We will descriptively analyze the weather characteristics captured by the 3D-printed weather stations. This includes assessing the occurrence of severe weather events, focusing on rainfall and heat events, and their potential impact on the health of participants, as measured by the wearable device and spirometer. For defining weather extremes, we will utilize the indices provided by the Expert Team on Climate Change Detection and Indices (ETCCDI) (58). Our analysis will encompass mean and min-max daily temperatures, as well as average and maximum daily wet bulb globe temperature (WBGT). We will also correlate sleep duration and night time heart rate with minimum night time temperature and the heat index, and step count with heavy rainfall events.

We will employ a linear mixed-effects model to explore the relationship between weather exposures and daily metrics like activity (steps), sleep, and nighttime heart rate. Covariates in the model will include the minimum nighttime heat index for sleep and nighttime HR, the maximum daytime WBGT for daily activities (steps), daily precipitation, type of day (weekend or workday), age group, gender, and body mass index (BMI) group of the participant. Additionally, we will investigate if patterns identified from the wearable device align with the self-reported activities of the participants.

We will also correlate lung function values, as determined by the spirometer at three distinct times for each cohort. This includes activity levels, sleep patterns, heart rate, and both indoor and outdoor temperature and humidity measurements, as well as the self-perceived burden of disease.

#### 2.9.2 Acceptability of sensor-based devices

We will analyze both qualitative (derived from IDIs, open-ended questions, and feedback sessions with stakeholders and field workers) and quantitative data (from Likert-scaled and other structured questionnaire items of the acceptability questionnaire, as well as sensor-based log data). This convergent approach seeks to offer a holistic understanding of the collected data. Descriptive analysis will be applied to responses from Likert-scaled and other structured questionnaire items. For open-ended questions, a thematic coding approach will be employed (59–61). Our analysis will adhere to the steps of “compiling, disassembling, reassembling, interpreting, and concluding” as outlined by (62).

#### 2.9.3 Feasibility of measuring the effects of heat stress

We will employ a two-way fixed effects regression (TWFE) to explore the relationships between weather-related variables, specifically heat and humidity, and lung function. Additionally, we will juxtapose these relationships with the correlations between weather-related variables and other indicators of heat stress, such as activity, sleep, and heart rate. By incorporating individual-level fixed effects, we aim to account for both observed and unobserved time-invariant individual confounders. Meanwhile, calendar time fixed effects, our ‘second method’ of fixed effects, will adjust for temporal changes shared across all study participants (63, 64). Recognizing that the impact of climate variables on lung function and other heat strain measures might differ, we will leverage recent advancements in TWFE analysis that yield unbiased estimates even amidst heterogeneous effect sizes (65, 66). For this analysis, we will utilize the DIDmultiplegt package in R (67).

### 2.10 Ethical considerations

The Heidelberg University Hospital Ethics Committee and MONASH University Ethics Committee granted ethical approval for this study in January 2023.

For details on informed consent, please refer to Supplementary material 2.

## 3 Results

We aim to establish the feasibility, reliability and validity of the sensor-based devices used within the SEACO HDSS. Metrics, including failure rate and data synchronization, will offer insight into the devices’ operational performance. By comparing data from 3D-printed weather stations with data of the Malaysian Meteorological Department, we seek to validate the accuracy and consistency of the weather measurements.

Our two-way fixed effects regression is anticipated to identify correlations between specific weather variables, including temperature and humidity, and lung function. Additionally, we anticipate to determine correlations between these weather variables and various measures of individual heat strain, such as activity levels, sleep patterns, and heart rate, which will provide insights into the direct and indirect impacts of weather variables on health outcomes.

Employing a mixed-methods approach, we expect to obtain qualitative data from participants, which will offer insights into experiences and perceptions regarding sensor-based devices. This feedback will be pivotal in guiding the optimal use of such devices at both individual and homebased levels, and refining the study design to leverage the wearable and indoor temperature and humidity measurement for future research for broader climate-health studies in larger populations.

With our data collection protocols and the digital devices employed, we expect a high degree of data completeness. Any gaps or inconsistencies in the data will be valuable in identifying areas for improvement in data collection methodologies.

Given the diverse demographic and cultural composition of the SEACO HDSS population, we anticipate diverse insights on how different subgroups perceive and experience the effects of heat. Such findings will be crucial for tailoring interventions and policies to meet specific community needs.

In conclusion, this study aims to delineate the potential of sensor-based technologies in capturing granular data that elucidates the nexus between weather variables and individual health impacts within the SEACO HDSS community. The outcomes will not only enrich the scientific discourse but also have potential implications for shaping public health strategies and interventions.

## 4 Discussion

In our study, we aim to identify optimal methods for integrating advanced data generation tools to enhance data granularity in climate change and health research, utilizing state-of-the-art sensor-based devices. We will also evaluate how such monitoring can seamlessly fit into the routine data collection of health and demographic surveillance sites. These devices will range from individual wearables tracking activity, sleep, heart rate, and lung function, to home-based instruments measuring indoor temperatures and humidity, and up to community-focused sensors like 3D-printed weather stations. A key objective will be to determine the prevalence of lung diseases sensitive and to understand impacts of heat, within the population.

Health and Demographic Surveillance Systems (HDSSs) are invaluable tools for research at the intersection of climate change and health. These systems provide a robust framework, collecting detailed data across large populations over extended durations. In over 56 Low- and Middle-Income Countries (LMICs) spanning Africa, Asia, and Oceania, HDSSs bridge the gap in population-health data, playing a crucial role in evidence-based decision-making (25, 68). Significantly, many of these LMICs with HDSSs bear the brunt of climate change impacts (69).

By enhancing the SEACO HDSS with additional climate metrics and health indicators, both self-reported and measured, we will likely be able to delve deeper into individual behaviors and exposures in their domestic and wider environments, supported by data from 3D-printed weather stations. This detailed data integration will elucidate individual exposures and their health implications, setting the stage for tailored population-level health interventions. Such a comprehensive dataset will likely offer a holistic view of health against the backdrop of climate change, deepening our grasp of the complex interplay between weather exposures and health outcomes.

The increasing frequency of severe and extreme weather events, driven by climate change, poses direct and indirect threats to human health, such as heat exposures and disruptions in food supply (5, 8, 69). Integrating sensor-based measurements into existing HDSSs offers a promising avenue for individual-level research, with the potential for scalability to encompass broader populations (70). In even resource-constrained environments, consumer-grade wearables can deliver detailed individual data, capturing metrics like activity, sleep, and vital signs (57). This continuous monitoring provides a detailed view of population movement and related exposures, such as heat, especially pertinent given the projected decrease in work capacity due to climate-related exposures (57). Analyzing correlations between wearable data and weather exposures can yield novel insights into the ongoing and long-term health impacts of climate change. Remote sensing techniques can enhance this approach by offering deeper insights, such as household crop yields, which can be correlated with individual data collected via wearables to understand more complex impacts on work capacity and nutritional outcomes. This method, leveraging satellite imagery, vegetation indices, and weather station data, may enhance our comprehension of nutritional security (71, 72).

Wearable devices have been effectively utilized in large-scale research to evaluate the health impacts of weather exposures related to climate change (73). Most existing studies predominantly target high-income countries, often neglecting populations already affected by climate-sensitive diseases, as underscored by Huhn et al. (74). Extreme heat poses risks in rural areas where residents often have limited financial resources, reside in inadequate housing, and lack air conditioning. The growing population of older adults is especially vulnerable due to common co-morbidities like cardiovascular diseases, reducing their heat resilience. Understanding the indoor exposures to heat and humidity, especially nighttime temperatures that influence sleep, is vital (75). Obradovich et al. highlighted that sleep disruptions from climate-driven nocturnal heat have pronounced physiological and psychological effects, especially on economically disadvantaged and older populations (75).

We aim to evaluate the feasibility of incorporating lung function evaluations into population health data collection. The burden of noncommunicable diseases (NCDs) seems to rise in the context of increased exposure to heat in many contexts, yet research remains limited in contexts, like Malaysia. However, there is limited research on the climate-related effects on NCDs, especially respiratory conditions like asthma and COPD, within the Malaysian context (76). The Spirobank spirometer, which we will use in our study, has received validation in several studies (77), including its effectiveness in early COPD detection (50). Lin et al. effectively employed the Spirobank spirometer to detect undiagnosed COPD cases within high-risk groups in Taiwan (50). This portable spirometer, integrated with an app, has been pivotal in identifying undiagnosed COPD in at-risk individuals (50).

3D-printed weather stations, priced at approximately $1,000 per unit, may offer a cost-effective alternative to professional weather stations, which can cost around $10,000 and often have to be imported. Professional weather stations not only come with high initial costs but also pose long-term maintenance challenges, especially in countries where spare parts are hard to come by. Additionally, there is the ever-present risk of these parts becoming obsolete. 3D-printed weather stations allow for local printing of replacement parts, promoting durability and sustainability. The capability to print multiple stations with a single 3D printer further cuts down on costs and fosters local ownership, as these stations can be printed and assembled on-site. The affordability and sustainability of 3D-printed weather stations might make them a compelling choice for enhancing HDSSs or similar research infrastructures. This could lead to a more extensive network of weather stations, yielding finer-grained weather data. Such detailed data is pivotal for climate change and health research, as well as for programs like weather index-based crop insurance, designed to alleviate the challenges farmers face from erratic weather patterns (78). Collecting detailed health and climate data to inform interventions is crucial. Our study aims to bolster this effort by generating robust climate and health data, enhancing the attractiveness of existing HDSSs for potential research funding.

We chose sensor-based devices, including wearables, spirometers, and home-based sensors, prioritizing a balance between quality and affordability. This approach ensures data reliability while considering scalability to encompass larger populations. Ease of data collection was also a primary factor in our selection. The devices we opted for, equipped with user-friendly interfaces and Bluetooth capabilities, streamline the data collection process. Our decision to use the Garmin wearable sensor was informed by its proven efficacy in population health studies in Burkina Faso and Kenya, as highlighted by Barteit et al. (70). The integration of the spirometer and home sensors was cost-effective and validated by their application in similar research settings.

## 5 Limitations

This study primarily serves as a feasibility assessment with a relatively small cohort of *n* = 120 participants. Such a limited sample size inherently limits the generalizability of our findings and the depth of insights for broader investigations. Furthermore, potential inaccuracies in sensor-based measurements should be acknowledged. Heart rate measurements might be affected by individual physiological variations, device fit, and the type and intensity of physical activity.

It is essential to highlight that the devices used in this study, including the spirometer and the wearable device (measuring lung function, heart rate, steps and sleep), are intended for recreational rather than medical purposes. They are not certified for diagnosing, monitoring, treating, or alleviating any medical condition or disease. Additionally, extended wear of the device might lead to skin discomfort, particularly for those with sensitive skin or allergies.

## Ethics statement

The studies involving humans were approved by Heidelberg University Hospital Ethics Committee and Monash University Malaysia. The studies were conducted in accordance with the local legislation and institutional requirements. Written informed consent for participation in this study was provided by the participants’ legal guardians/next of kin. Written informed consent was obtained from the individual(s) for the publication of any potentially identifiable images or data included in this article.

## Author contributions

SB wrote the first draft of the study protocol with the assistance of DC. The initial study design was developed by TB, TS, and SB. SN, MT, DG, and DC contributed to the development of the detailed study design. Data collection and local study management are locally managed and conducted by SN, MT, TS, and DG supervised by TS. The acquired data will be statistically analyzed by SN, MT, and SB. All authors contributed to the article and approved the submitted version.
