# Supplementary material for: Developing Climate Change and Health Impact Monitoring with eHealth at the South East Asia Community Observatory and Health and Demographic Surveillance Site, Malaysia (CHIMES)
Source: Front Public Health. 2023 Dec 1;11:1153149. doi: 10.3389/fpubh.2023.1153149 (PMC10732307; doi:10.3389/fpubh.2023.1153149)
Supplement: Supplementary file 1 [file Data_Sheet_1.PDF]

Generated by cp1, Dec 07, 2022 22:38
Questionnaire created by cp1, Jul 12, 2022 17:19
Last modified by cp1, Nov 01, 2022 15:20

Shared with:
Syahrul (never edited)

Sections: 8, Sub-sections: 2,
Questions: 123.
Questions with enabling conditions: 25
Questions with validation conditions:1
Rosters: 2
Variables: 2

Survey Solutions

CHIMES feasibility study

SURVEY IDENTIFICATION INFORMATION
QUESTIONNAIRE DESCRIPTION

STUDY PARTICIPANT'S DETAILS
No sub-sections, No rosters, Questions: 7, Static texts: 1.

PRE-SCREENING QUESTIONNAIRE OF OBSTRUCTIVE LUNG DISEASES
Sub-sections: 2, No rosters, Questions: 12, Static texts: 2, Variables: 2.

DISTRIBUTION/ RETURN OF DEVICE
No sub-sections, Rosters: 2, Questions: 28, Static texts: 1.

ACCEPTANCE QUESTIONNAIRE
No sub-sections, No rosters, Questions: 35, Static texts: 2.

ACTIVITY DIARY
No sub-sections, No rosters, Questions: 21.

HEAT QUESTIONNAIRE
No sub-sections, No rosters, Questions: 5.

BURDEN OF RESPIRATORY DISEASE QUESTIONNAIRE
No sub-sections, No rosters, Questions: 8.

3D-PRINTED WEATHER STATION
No sub-sections, No rosters, Questions: 7, Static texts: 1.

APPENDIX A — CATEGORIES

LEGEND

SURVEY IDENTIFICATION INFORMATION QUESTIONNAIRE DESCRIPTION

Basic information

Title CHIMES feasibility study

Survey data information

Study type Integrated Survey

Kind of data Aggregate data [agg]

Survey information

Country Malaysia

Year 2022

Languages English

STUDY PARTICIPANT'S DETAILS

study_part_details

STATIC TEXT

Study procedure: --- 1. Take the participant's device --- --- 2. Disinfect the participant's device --- --- 3. Put the device on the charger --- --- 4. Synchronize the data of the device with the study participant's profile by using the app on the tablet --- --- 5. Fill in the section "Distribution/return of device" --- --- 6. Take the participant's blood pressure measurement and record it in the Health Mate application --- --- 7. Ask the questions of the acceptance questionnaire --- --- 8. Ask the questions of the activity diary questionnaire ---

Name of participant's village	SINGLE-SELECT: COMBO BOX nom_district<br>01 O Jabi<br>02 O Sunga Segamat<br>03 O Gemerek<br>04 O Bekok<br>05 O Chaah
Location ID of participant's HDSS residence	TEXT locationid<br>..
Household ID	TEXT idhh<br>..
Enter the participant's study ID<br>I The participant's study ID has 14 positions.<br>V1 `Regex.IsMatch(self,"^\\d{1,3}-\\d{1,3}-\\d{1,3}-\\d{1,3}$") || Regex.IsMatch(self,"Q")`	TEXT study_part_id<br>..
Sex assigned at birth of participant	SINGLE-SELECT sex<br>01 O Male<br>02 O Female
Is the Participant part of the Subsample COPD/Asthma?	SINGLE-SELECT subsample<br>01 O Yes<br>00 O No
Do you have any other medical pre-condition besides Asthma or COPD? For example High blood pressure, Diabetes, Depression or else? Please name:	TEXT pre_condition<br>..

PRE-SCREENING QUESTIONNAIRE OF OBSTRUCTIVE LUNG DISEASES

prescree_old

Date	DATE: CURRENT TIME date_old<br>..

PRE-SCREENING QUESTIONNAIRE OF OBSTRUCTIVE LUNG DISEASES

COULD IT BE COPD?-QUESTIONNAIRE

COPD_questionnaire

Do you cough several times most days?	SINGLE-SELECT cough_most_days<br>00 ○ No<br>01 ○ Yes
Do you bring up phlegm or mucus most days?	SINGLE-SELECT phlegm_most_days<br>00 ○ No<br>01 ○ Yes
Do you get out of breath more easily than othe rs your age?	SINGLE-SELECT out_of_breath<br>00 ○ No<br>01 ○ Yes
Are you older than 40 years?	SINGLE-SELECT age_above_40<br>00 ○ No<br>01 ○ Yes
Are you a current smoker or an ex-smoker?	SINGLE-SELECT current_ex_smoker<br>00 ○ No<br>01 ○ Yes
VARIABLE<br>(cough_most_days+phlegm_most_days+out_of_breath+age_above_40+current_ex_smoker)	DOUBLE copd_score

STATIC TEXT

E copd_score >= 3

Please conduct a spirometry test with the study participant (take at least 3 measurements and store data within the Spirobank Smart app).

PRE-SCREENING QUESTIONNAIRE OF OBSTRUCTIVE LUNG DISEASES

ASTHMA SCREENING QUESTIONNAIRE (ASQ)

Do you cough more than the average person?	SINGLE-SELECT cough_more<br>02 ○ Yes<br>00 ○ No
Do you have a cough that comes mainly from y our chest and NOT from your throat?	SINGLE-SELECT cough_chest<br>02 ○ Yes<br>00 ○ No

Question	Response
Do you have worsening of the following sympt oms when you lie down to sleep?	MULTI-SELECT sleep_symptoms<br>01 ☐ Cough<br>02 ☐ Chest tightness<br>03 ☐ Wheeze<br>04 ☐ Shortness of breath
Do you have worsening of the following sympt oms after exercise or physical activity?	MULTI-SELECT physical_symptoms<br>01 ☐ Cough<br>02 ☐ Chest tightness<br>03 ☐ Wheeze<br>04 ☐ Shortness of breath
Do you have worsening of the following sympt oms after laughing or crying?	MULTI-SELECT laugh_cry_symptoms<br>01 ☐ Cough<br>02 ☐ Chest tightness<br>03 ☐ Wheeze<br>04 ☐ Shortness of breath
Do you have worsening of the following sympt oms after talking on the phone?	MULTI-SELECT phone_symptoms<br>01 ☐ Cough<br>02 ☐ Chest tightness<br>03 ☐ Wheeze<br>04 ☐ Shortness of breath
VARIABLE<br>`(cough_more+cough_chest+sleep_symptoms.Sum()+physical_symptoms.Sum()+laugh_cry_symptoms.Sum()+phone_symptoms.Sum())`	LONG asthma_score

STATIC TEXT

E `asthma_score >= 4`

Please conduct a spirometry test with the study participant (take at least 3 measurements and store data within the Spirobank Smart app).

DISTRIBUTION/ RETURN OF DEVICE

sensor_exchange

Question	Answer
Would you like to distribute or to return a devic e?	SINGLE-SELECT return_handout_sensor<br>01 ○ Distribution - I would like to start the distribution of a de vice.<br>02 ○ Return - I would like to start the return of a device.
Which device has been distributed?<br>E return_handout_sensor==1	MULTI-SELECT sensor_type_distrib<br>01 ☐ Garmin VivoSmart 5 (black wristband)<br>02 ☐ None

DISTRIBUTION/ RETURN OF DEVICE

Roster: WHICH DEVICE HAS BEEN DISTRIBUTED? - %ROSTERTITLE%

generated by multi-select question sensor_type_distrib

roster_1_sensor_distributed

E return_handout_sensor==1

Question	Answer
Which number has the distributed device %ros tertitle%?<br>E @rowcode==1	SINGLE-SELECT: COMBO BOX garmin_num_sensor_distributed<br>001 ○ GL-3104019<br>002 ○ GL-3104020<br>003 ○ GL-3104021<br>004 ○ GL-3104022<br>005 ○ GL-3104023<br>006 ○ GL-3104024<br>007 ○ GL-3104025<br>008 ○ GL-3104026<br>009 ○ GL-3104027<br>010 ○ GL-3104028<br>011 ○ GL-3104029<br>012 ○ GL-3104030<br>013 ○ GL-3104031<br>014 ○ GL-3104032<br>015 ○ GL-3104033<br>016 ○ GL-3104034<br>And 84 other symbols [3]
Please note the distribution date of the device %rostertitle%.<br>I Please fill in the date the following way: day (dd) - month (mm) - year (y y)	DATE sensor_date_distributed<br>..

Question	Response
Is the device %rostertitle% disinfected?	SINGLE-SELECT sensor_desinfected<br>01 ○ Yes, it is disinfected.<br>02 ○ No, it isn't disinfected.<br>03 ○ Comment

STATIC TEXT

E sensor_desinfected==2

Warning! Please sanitize the device before proceeding.

Question	Response
Comment (disinfection of the device)<br>E sensor_desinfected==3	TEXT sensor_desinfected_comm<br>..
The study participant is registered in the app o n the tablet for the device %rostertitle%?	SINGLE-SELECT registration_stud_part_app<br>01 ○ Yes, I have registered the st udy participant in the app.<br>02 ○ No, I haven't registered the study participant in the app.<br>03 ○ Comment
Comment (registration)<br>E registration_stud_part_app==3	TEXT registration_stud_part_app_comm<br>..
Are you comfortable with the device's %rostert itle% tablet application?	SINGLE-SELECT ease_app_distribution<br>01 ○ Strongly agree<br>02 ○ Agree<br>03 ○ Neither agree nor disagree<br>04 ○ Disagree<br>05 ○ Strongly disagree
Has it been easy for you to synchronize the dat a of the device with the study participant?	SINGLE-SELECT difficulty_usage_app_distributi<br>01 ○ Strongly agree<br>02 ○ Agree<br>03 ○ Neither agree nor disagree<br>04 ○ Disagree<br>05 ○ Strongly disagree
It has been easy for you to manage the study p articipant with the tablet.	SINGLE-SELECT knowledge_level_app_distribution<br>01 ○ Strongly agree<br>02 ○ Agree<br>03 ○ Neither agree nor disagree<br>04 ○ Disagree<br>05 ○ Strongly disagree

The participant is satisfied with the device %rostertitle%.	SINGLE-SELECT	feedback_stud_part_distribution
	01	○ Strongly agree
	02	○ Agree
	03	○ Neither agree nor disagree
	04	○ Disagree
	05	○ Strongly disagree
The participant accepted the device %rostertitle%.	SINGLE-SELECT	acceptance_stud_part_distributio
	01	○ Strongly agree
	02	○ Agree
	03	○ Neither agree nor disagree
	04	○ Disagree
	05	○ Strongly disagree
The participant was happy to wear the device %rostertitle%.	SINGLE-SELECT	happy_stud_part_distribution
	01	○ Strongly agree
	02	○ Agree
	03	○ Neither agree nor disagree
	04	○ Disagree
	05	○ Strongly disagree
The participant is frustrated by the device %rostertitle%.	SINGLE-SELECT	frustration_stud_part_distributi
	01	○ Strongly agree
	02	○ Agree
	03	○ Neither agree nor disagree
	04	○ Disagree
	05	○ Strongly disagree

Which device has been returned?	MULTI-SELECT	sensor_type_returned
E return_handout_sensor==2	01	☐ Garmin VivoSmart 5 (black wristband)
	02	☐ None

DISTRIBUTION/ RETURN OF DEVICE

Roster: WHICH DEVICE HAS BEEN RETURNED? - %ROSTERTITLE%

generated by multi-select question sensor_type_returned

roster_2_sensor_returned

E return_handout_sensor==2

Which number has the returned device %rostertitle%?<br><br>E @rowcode==1	SINGLE-SELECT: COMBO BOX garmin_num_sensor_returned<br>001 GL-3104019<br>002 GL-3104020<br>003 GL-3104021<br>004 GL-3104022<br>005 GL-3104023<br>006 GL-3104024<br>007 GL-3104025<br>008 GL-3104026<br>009 GL-3104027<br>010 GL-3104028<br>011 GL-3104029<br>012 GL-3104030<br>013 GL-3104031<br>014 GL-3104032<br>015 GL-3104033<br>016 GL-3104034<br><br>And 84 other symbols [3]
Please enter the return date of the device %rostertitle%.<br><br>I Please fill in the date the following way: day (dd) - month (mm) - year (yy)	DATE sensor_date_returned<br><br>..
Is the device defect?	SINGLE-SELECT sensor_status_returned<br>01 Yes, it is defect.<br>02 No, it works.<br>03 Other
Other (device %rostertitle% defect)<br><br>E sensor_status_returned==3	TEXT comment_sensor_status_returned<br><br>..
Are you comfortable with the device's %rostertitle% tablet application?	SINGLE-SELECT ease_usage_app<br>01 Strongly agree<br>02 Agree<br>03 Neither agree nor disagree<br>04 Disagree<br>05 Strongly disagree
It has been easy for you to synchronize the data of the study participant's device.	SINGLE-SELECT difficulty_usage_app<br>01 Strongly agree<br>02 Agree<br>03 Neither agree nor disagree<br>04 Disagree<br>05 Strongly disagree

It has been easy for you to manage the study participant with the tablet.	SINGLE-SELECT knowledge_level_app<br>01 ◯ Strongly agree<br>02 ◯ Agree<br>03 ◯ Neither agree nor disagree<br>04 ◯ Disagree<br>05 ◯ Strongly disagree
The participant is satisfied with the device %rostertitle%.	SINGLE-SELECT feedback_stud_part<br>01 ◯ Strongly agree<br>02 ◯ Agree<br>03 ◯ Neither agree nor disagree<br>04 ◯ Disagree<br>05 ◯ Strongly disagree
The participant accepted the device %rostertitle%.	SINGLE-SELECT acceptance_stud_part<br>01 ◯ Strongly agree<br>02 ◯ Agree<br>03 ◯ Neither agree nor disagree<br>04 ◯ Disagree<br>05 ◯ Strongly disagree
The participant was happy to wear the device %rostertitle%.	SINGLE-SELECT happy_stud_part<br>01 ◯ Strongly agree<br>02 ◯ Agree<br>03 ◯ Neither agree nor disagree<br>04 ◯ Disagree<br>05 ◯ Strongly disagree
The participant is frustrated by the device %rostertitle%.	SINGLE-SELECT frustration_stud_part<br>01 ◯ Strongly agree<br>02 ◯ Agree<br>03 ◯ Neither agree nor disagree<br>04 ◯ Disagree<br>05 ◯ Strongly disagree
Other comments	TEXT general_comments_sensor_return<br>..

ACCEPTANCE QUESTIONNAIRE

STATIC TEXT

Bevor starting the interview with the study participant, please put the (s) device (s) on the charging station. Please ask the study participant all of the questions. Thank you for your accurate collaboration.

I am happy to use the device.	SINGLE-SELECT positive_att_sensor<br>01 ○ Strongly agree<br>02 ○ Agree<br>03 ○ Neither agree nor disagree<br>04 ○ Disagree<br>05 ○ Strongly disagree
I am upset using the device	SINGLE-SELECT disturbed_sensor<br>01 ○ Strongly agree<br>02 ○ Agree<br>03 ○ Neither agree nor disagree<br>04 ○ Disagree<br>05 ○ Strongly disagree
Are you wearing more than one device?	SINGLE-SELECT multiple_sensors<br>01 ○ Yes<br>02 ○ No
Comment (type of device) - please describe<br>E multiple_sensors==1	TEXT multiple_sensors_type<br>..
It has been easy for me to wear the device this week.	SINGLE-SELECT week_experience1_sensor<br>01 ○ Strongly agree<br>02 ○ Agree<br>03 ○ Neither agree nor disagree<br>04 ○ Disagree<br>05 ○ Strongly disagree
It has been uncomfortable for me to wear the device this week.	SINGLE-SELECT week_experience2_sensor<br>01 ○ Strongly agree<br>02 ○ Agree<br>03 ○ Neither agree nor disagree<br>04 ○ Disagree<br>05 ○ Strongly disagree

Question	Response
What have you liked/ disliked about the device? (multiple choice)	MULTI-SELECT: ORDERED `like_dislike_sensor`<br>01 ☐ easy to wear<br>02 ☐ good weight<br>03 ☐ good handiness<br>04 ☐ nice appearance<br>05 ☐ practical to wear<br>06 ☐ comfortable to wear<br>07 ☐ too bulky<br>08 ☐ too heavy<br>09 ☐ too big<br>10 ☐ difficult to wear<br>11 ☐ Other (please describe)
Comment (like/ dislike) - please describe	TEXT `like_dislike_sensor_comment`<br>..
Did you have any problems with the device this week?	SINGLE-SELECT `challenges_sensor`<br>01 ○ Yes<br>02 ○ No
This week's problems with the device. (multiple choice)<br>E `challenges_sensor==1`	MULTI-SELECT: ORDERED `challenges_sensor_y`<br>01 ☐ itchy skin<br>02 ☐ itch<br>03 ☐ the device caused pain<br>04 ☐ it limited my movements<br>05 ☐ it was disturbing during wor k<br>06 ☐ it was disturbing during slee p<br>07 ☐ it was disturbing the daily ro utine<br>08 ☐ electric shock<br>09 ☐ Other (please describe)
Comment (this week's problems with the devic e) - please describe	TEXT `challenges_sensor_yes_comments`<br>..
Wearing the device affected my work/ my daily activities.	SINGLE-SELECT `daily_life_sensor`<br>01 ○ Strongly agree<br>02 ○ Agree<br>03 ○ Neither agree nor disagree<br>04 ○ Disagree<br>05 ○ Strongly disagree

Question	Response
How did it feel to wear the device? (multiple ch oice)	MULTI-SELECT: ORDERED — reactions_sensor<br>01 ☐ I forgot that I was wearing it .<br>02 ☐ I have not been disturbed.<br>03 ☐ Sometimes difficult<br>04 ☐ it needed time/ attention<br>05 ☐ I have interrupted my activit ies several times because of the device.<br>06 ☐ I had to remove the device<br>07 ☐ The device limited my move ments<br>08 ☐ I had pain or other undesire d effects (like a skin rash, itc hing)<br>09 ☐ The sensor was stuck to my skin because of the sweat.<br>10 ☐ I felt an increase in heat fro m wearing the sensor.<br>11 ☐ Other (please describe)
Comment (device's effects) - please describe	TEXT — reactions_sensor_comment<br>..
Has wearing the device had effects on your sle ep?	SINGLE-SELECT — sleep_sensor<br>01 ○ Yes<br>02 ○ No
Wearing the device had the following effects o n my sleep... (multiple choice)<br>E sleep_sensor==1	MULTI-SELECT: ORDERED — sleep_sensor_2<br>01 ☐ I woke up sometimes.<br>02 ☐ I woke up frequently.<br>03 ☐ I couldn't sleep at all becaus e of the device.<br>04 ☐ I felt tired in the morning (p oor sleep quality).<br>05 ☐ The device fell off me during the night.<br>06 ☐ I felt an increase in heat fro m wearing the device.<br>07 ☐ Other (please describe)
Comments (device's effects on my sleep) - plea se describe	TEXT — sleep_sensor_3<br>..

Question	Response options	Variable
Did you have to remove the device?	SINGLE-SELECT<br>01 ○ Yes<br>02 ○ No	removing_sensor
I removed the device because... (multiple choice)<br>E removing_sensor==1	MULTI-SELECT: ORDERED, YES/NO<br>01 ☐/☐ It was limiting my activities<br>02 ☐/☐ I had undesired effects<br>03 ☐/☐ Other (please describe)	removing_sensor_2
Comment (removement of the device) - please describe	TEXT<br>..	removing_sensor_3
I was comfortable wearing the device in public	SINGLE-SELECT<br>01 ○ Strongly agree<br>02 ○ Agree<br>03 ○ Neither agree nor disagree<br>04 ○ Disagree<br>05 ○ Strongly disagree	sensor_public
Have people asked you about the device?	SINGLE-SELECT<br>01 ○ Yes<br>02 ○ No	sensor_public_2
On which of the devices have you been asked questions?<br>E sensor_public_2 == 1	MULTI-SELECT<br>01 ☐ Garmin VivoSmart 5 (black wristband)<br>02 ☐ None	sensor_public_2_1
Please describe, what people have asked you about the device.<br>E sensor_public_2 == 1	TEXT<br>..	sensor_public_2_2
If you had to wear this device for a longer period of time (i.e. one year), what would be the barriers for you to participate in such a study (multiple choice)	MULTI-SELECT<br>01 ☐ Time required to wear<br>02 ☐ time spent participating in studies<br>03 ☐ the sensor does not give me any information about my health<br>04 ☐ Undesired effects<br>05 ☐ Social acceptance<br>06 ☐ Disturbance of daily activities<br>07 ☐ Disturbance of my sleep<br>08 ☐ Disturbance of my personal hygiene routine<br>09 ☐ Other (please describe)	longterm_sensor

Comment (barriers of a long-term device study) - please describe	TEXT longterm_sensor_comment<br>..

STATIC TEXT

The next questions refer to the home-based sensor "SwitchBot Meter". Please inform the study participant that the next questions are about the sensor in their homes, the so-called "SwitchBot Meter"

I am happy to have the device in my home.	TEXT positive_att_sensor_homebased<br>..
I am not comfortable to have the device in my home.	SINGLE-SELECT negative_att_sensor_homebased<br>01 ○ Strongly agree<br>02 ○ Agree<br>03 ○ Neither agree nor disagree<br>04 ○ Disagree<br>05 ○ Strongly disagree
Did you have problems with the homebased se nsor this week (SwitchBot Meter)?	SINGLE-SELECT challenges_sensor_homebased<br>01 ○ Strongly agree<br>02 ○ Agree<br>03 ○ Neither agree nor disagree<br>04 ○ Disagree<br>05 ○ Strongly disagree
What have you liked/disliked about having the sensor in your home?	TEXT like_dislike_sensor_home_com<br>..
Have people asked you about the device in you r home?	SINGLE-SELECT homebased_sensor_public<br>01 ○ Yes<br>02 ○ No
Please describe, what people have asked you a bout the device in your home.<br>E homebased_sensor_public == 1	TEXT homebased_sensor_public_2<br>..
Did you have to remove the device in your hom e?	SINGLE-SELECT removing_hb_sensor<br>01 ○ Yes<br>02 ○ No
I removed the device in my hoome because... (multiple choice)<br>E removing_hb_sensor==1	MULTI-SELECT: ORDERED, YES/NO removing_hb_sensor_2<br>01 ☐/☐ It was limiting my activ ities<br>02 ☐/☐ I had undesired effects<br>03 ☐/☐ Other (please describe)
Comment (removal of the device in your home) - please describe	TEXT removing_hb_sensor_3<br>..

ACTIVITY DIARY

Question	Answer
What activity did you do after you got up in the morning? (multiple choice)	MULTI-SELECT: ORDERED `after_gettingup`<br>01 ☐ working on the farm/field/harvesting<br>02 ☐ cooking<br>03 ☐ animal husbandry<br>04 ☐ taking care of children<br>05 ☐ taking care of family members<br>06 ☐ getting water from the well<br>07 ☐ going to school<br>08 ☐ doing sport<br>09 ☐ selling products at the market<br>10 ☐ buying<br>11 ☐ I rested<br>12 ☐ watching TV<br>13 ☐ going to a bar/restaurant<br>14 ☐ working in a sitting position inside/office work<br>15 ☐ working in a sitting position outside<br>16 ☐ working by hand/manual/casual work<br>And 3 other symbols [5]
Did you take any protective measures against the heat?	SINGLE-SELECT `protective_heat_gettinup`<br>01 ○ Yes<br>00 ○ No

Which measures did you take against the heat?

E protective_heat_gettinup==1

MULTI-SELECT protective_m_gu

01 ☐ Staying in the shade
02 ☐ Drinking more water
03 ☐ Drinking cool beverages other than water
04 ☐ Using a hat/ sunscreen
05 ☐ Staying inside
06 ☐ Leaving doors/windows open for ventilation
07 ☐ Using airconditioning
08 ☐ Going to a public place with airconditioning
09 ☐ Bathing/showering frequently
10 ☐ Resting
11 ☐ Using shades or blinds to keep the house cool
12 ☐ Other

What activity did you do in the morning? (multiple choice)

MULTI-SELECT morning_activity

01 ☐ working on the farm/field/harvesting
02 ☐ cooking
03 ☐ animal husbandry
04 ☐ taking care of children
05 ☐ taking care of family members
06 ☐ getting water from the well
07 ☐ going to school
08 ☐ doing sport
09 ☐ selling products at the market
10 ☐ buying
11 ☐ I rested
12 ☐ watching TV
13 ☐ going to a bar/restaurant
14 ☐ working in a sitting position inside/office work
15 ☐ working in a sitting position outside
16 ☐ working by hand/manual/casual work

And 3 other symbols [5]

Question	Type	Code	Answer	Variable
Did you take any protective measures against t he heat?	SINGLE-SELECT	01	◯ Yes	protective_heat_morning
		00	◯ No	
Which measures did you take against the heat?<br>E protective_heat_morning==1	MULTI-SELECT	01	☐ Staying in the shade	protective_m_mo
		02	☐ Drinking more water	
		03	☐ Drinking cool beverages oth er than water	
		04	☐ Using a hat/ sunscreen	
		05	☐ Staying inside	
		06	☐ Leaving doors/windows ope n for ventilation	
		07	☐ Using airconditioning	
		08	☐ Going to a public place with airconditioning	
		09	☐ Bathing/showering frequen tly	
		10	☐ Resting	
		11	☐ Using shades or blinds to ke ep the house cool	
		12	☐ Other	

What activity did you do in the midday? (multiple choice)	MULTI-SELECT noon_activity<br>01 ☐ working on the farm/field/harvesting<br>02 ☐ cooking<br>03 ☐ animal husbandry<br>04 ☐ taking care of children<br>05 ☐ taking care of family members<br>06 ☐ getting water from the well<br>07 ☐ going to school<br>08 ☐ doing sport<br>09 ☐ selling products at the market<br>10 ☐ buying<br>11 ☐ I rested<br>12 ☐ watching TV<br>13 ☐ going to a bar/restaurant<br>14 ☐ working in a sitting position inside/office work<br>15 ☐ working in a sitting position outside<br>16 ☐ working by hand/manual/casual work<br><br>And 3 other symbols [5]
Did you take any protective measures against the heat?	SINGLE-SELECT protective_heat_noon<br>01 ○ Yes<br>00 ○ No

Which measures did you take against the heat?	MULTI-SELECT protective_m_no
E protective_heat_noon==1	01 ☐ Staying in the shade<br>02 ☐ Drinking more water<br>03 ☐ Drinking cool beverages other than water<br>04 ☐ Using a hat/ sunscreen<br>05 ☐ Staying inside<br>06 ☐ Leaving doors/windows open for ventilation<br>07 ☐ Using airconditioning<br>08 ☐ Going to a public place with airconditioning<br>09 ☐ Bathing/showering frequently<br>10 ☐ Resting<br>11 ☐ Using shades or blinds to keep the house cool<br>12 ☐ Other
What activity did you do in the afternoon? (multiple choice)	MULTI-SELECT afternoon_activity<br>01 ☐ working on the farm/field/harvesting<br>02 ☐ cooking<br>03 ☐ animal husbandry<br>04 ☐ taking care of children<br>05 ☐ taking care of family members<br>06 ☐ getting water from the well<br>07 ☐ going to school<br>08 ☐ doing sport<br>09 ☐ selling products at the market<br>10 ☐ buying<br>11 ☐ I rested<br>12 ☐ watching TV<br>13 ☐ going to a bar/restaurant<br>14 ☐ working in a sitting position inside/office work<br>15 ☐ working in a sitting position outside<br>16 ☐ working by hand/manual/casual work<br><br>And 3 other symbols [5]

Did you take any protective measures against t he heat?	SINGLE-SELECT<br>01 ○ Yes<br>00 ○ No	protective_heat_afternoon
Which measures did you take against the heat?<br>E protective_heat_afternoon==1	MULTI-SELECT<br>01 ☐ Staying in the shade<br>02 ☐ Drinking more water<br>03 ☐ Drinking cool beverages oth er than water<br>04 ☐ Using a hat/ sunscreen<br>05 ☐ Staying inside<br>06 ☐ Leaving doors/windows ope n for ventilation<br>07 ☐ Using airconditioning<br>08 ☐ Going to a public place with airconditioning<br>09 ☐ Bathing/showering frequen tly<br>10 ☐ Resting<br>11 ☐ Using shades or blinds to ke ep the house cool<br>12 ☐ Other	protective_m_ano

What activity did you do in the evening? (multip le choice)	MULTI-SELECT evening_activity<br>01 ☐ working on the farm/field/harvesting<br>02 ☐ cooking<br>03 ☐ animal husbandry<br>04 ☐ taking care of children<br>05 ☐ taking care of family members<br>06 ☐ getting water from the well<br>07 ☐ going to school<br>08 ☐ doing sport<br>09 ☐ selling products at the market<br>10 ☐ buying<br>11 ☐ I rested<br>12 ☐ watching TV<br>13 ☐ going to a bar/restaurant<br>14 ☐ working in a sitting position inside/office work<br>15 ☐ working in a sitting position outside<br>16 ☐ working by hand/manual/casual work<br>And 3 other symbols [5]
Did you take any protective measures against t he heat?	SINGLE-SELECT protective_heat_evening<br>01 ○ Yes<br>00 ○ No

Question	Options
Which measures did you take against the heat?<br>E protective_heat_evening==1	MULTI-SELECT protective_m_ev<br>01 ☐ Staying in the shade<br>02 ☐ Drinking more water<br>03 ☐ Drinking cool beverages oth er than water<br>04 ☐ Using a hat/ sunscreen<br>05 ☐ Staying inside<br>06 ☐ Leaving doors/windows ope n for ventilation<br>07 ☐ Using airconditioning<br>08 ☐ Going to a public place with airconditioning<br>09 ☐ Bathing/showering frequen tly<br>10 ☐ Resting<br>11 ☐ Using shades or blinds to ke ep the house cool<br>12 ☐ Other
What activity did you do in the night? (multiple choice)	MULTI-SELECT night_activity<br>01 ☐ working on the farm/field/h arvesting<br>02 ☐ cooking<br>03 ☐ animal husbandry<br>04 ☐ taking care of children<br>05 ☐ taking care of family memb ers<br>06 ☐ getting water from the well<br>07 ☐ going to school<br>08 ☐ doing sport<br>09 ☐ selling products at the mark et<br>10 ☐ buying<br>11 ☐ I rested<br>12 ☐ watching TV<br>13 ☐ going to a bar/restaurant<br>14 ☐ working in a sitting position inside/office work<br>15 ☐ working in a sitting position outside<br>16 ☐ working by hand/manual/ca sual work<br>And 3 other symbols [5]

Did you take any protective measures against t he heat?	SINGLE-SELECT protective_heat_night<br>01 ○ Yes<br>00 ○ No
Which measures did you take against the heat?<br>E protective_heat_night==1	MULTI-SELECT protective_m_ni<br>01 ☐ Staying in the shade<br>02 ☐ Drinking more water<br>03 ☐ Drinking cool beverages oth er than water<br>04 ☐ Using a hat/ sunscreen<br>05 ☐ Staying inside<br>06 ☐ Leaving doors/windows ope n for ventilation<br>07 ☐ Using airconditioning<br>08 ☐ Going to a public place with airconditioning<br>09 ☐ Bathing/showering frequen tly<br>10 ☐ Resting<br>11 ☐ Using shades or blinds to ke ep the house cool<br>12 ☐ Other
Please add the activity (and the time of the day) and describe it, if possible.	LIST comment_activity<br>..
Did you take any protective measures against t he heat?	SINGLE-SELECT protective_heat_comment<br>01 ○ Yes<br>00 ○ No

Which measures did you take against the heat?<br>E protective_heat_comment==1	MULTI-SELECT protective_m_com<br>01 ☐ Staying in the shade<br>02 ☐ Drinking more water<br>03 ☐ Drinking cool beverages oth er than water<br>04 ☐ Using a hat/ sunscreen<br>05 ☐ Staying inside<br>06 ☐ Leaving doors/windows ope n for ventilation<br>07 ☐ Using airconditioning<br>08 ☐ Going to a public place with airconditioning<br>09 ☐ Bathing/showering frequen tly<br>10 ☐ Resting<br>11 ☐ Using shades or blinds to ke ep the house cool<br>12 ☐ Other

HEAT QUESTIONNAIRE

Question	Response
Do you consider heat to be a problem in your d aily life?	SINGLE-SELECT heat_problem<br>00 ○ No<br>01 ○ Yes
In what situation does it impact you the most?<br>E heat_problem ==1	MULTI-SELECT heat_situations<br>01 ☐ During the night<br>02 ☐ During the morning<br>03 ☐ During midday<br>04 ☐ During the afternoon<br>05 ☐ During the evening<br>06 ☐ During work outside<br>07 ☐ During work inside the hous e<br>08 ☐ Drinkwater shortage<br>09 ☐ Other
In what other situation does heat impact you. P lease name:<br>E heat_problem == 1	TEXT heat_situation_other<br>..
Are there other environmental/weather influen ces that negatively affect you? (e.g., flooding d uring heavy rains)	SINGLE-SELECT heat_environmental<br>00 ○ No<br>01 ○ Yes
Please describe the environmental/weather co nditions that affect you negatively<br>E heat_environmental == 1	TEXT heat_environmental_specific<br>..

E subsample==1

Question	Responses
On average, during the past week, how often d id you feel short of breath at rest?	SINGLE-SELECT short_breath_rest<br>01 Never<br>02 Hardly ever<br>03 A few times<br>04 Several times<br>05 Many times<br>06 A great many times<br>07 Almost all the time
On average, during the past week, how often d id you feel short of breath doing physical activi ties?	SINGLE-SELECT short_breath_physical<br>01 Never<br>02 Hardly ever<br>03 A few times<br>04 Several times<br>05 Many times<br>06 A great many times<br>07 Almost all the time
On average, during the past week, how often d id you feel short of breath because of heat?	SINGLE-SELECT short_breath_heat<br>01 Never<br>02 Hardly ever<br>03 A few times<br>04 Several times<br>05 Many times<br>06 A great many times<br>07 Almost all the time
On average, during the past week, how limited were you in doing Strenuous physical activities (such as climbing stairs, hurrying, doing sports), because of your breathing problems?	SINGLE-SELECT limitation_strenous<br>01 Not limited at all<br>02 Very slightly limited<br>03 Slightly limited<br>04 Moderately limited<br>05 Very limited<br>06 Extremely limited<br>07 Totally limited or unable to do so

Question	Response
On average, during the past week, how limited were you in doing daily activities at home (such as dressing, washing yourself) because of your breathing problems?	SINGLE-SELECT `limitation_daily`<br>01 Not limited at all<br>02 Very slightly limited<br>03 Slightly limited<br>04 Moderately limited<br>05 Very limited<br>06 Extremely limited<br>07 Totally limited or unable to do so
On average, during the past week, how often d id you feel limited in doing physical activities be cause of heat?	SINGLE-SELECT `short_physical_heat`<br>01 Never<br>02 Hardly ever<br>03 A few times<br>04 Several times<br>05 Many times<br>06 A great many times<br>07 Almost all the time
How often in the past week did you suffer fro m worry about your respiratory disease and its implications on your daily life?	SINGLE-SELECT `worries`<br>01 Never<br>02 Hardly ever<br>03 A few times<br>04 Several times<br>05 Many times<br>06 A great many times<br>07 Almost all the time
On average, during the past week, how often d id you feel tired because of heat?	SINGLE-SELECT `short_tired_heat`<br>01 Never<br>02 Hardly ever<br>03 A few times<br>04 Several times<br>05 Many times<br>06 A great many times<br>07 Almost all the time

3D-PRINTED WEATHER STATION

STATIC TEXT

This section is only relevant for field workers who inspect the weather station.

When have you done the inspection of the wea ther station?	DATE date_inspection
Where is the weather station located?	GEOGRAPHY location_ws
Have you noticed any visible damages when ins pecting the weather station?	SINGLE-SELECT damage_ws<br>00 ○ No<br>01 ○ Yes
Please take pictures of the damages.	PICTURE picture_damages_ws
Please note down any comments regarding th e weather station damages.	TEXT comments_damages
What have persons in the surrounding areas s aid about the weather station?	TEXT feedback_ws
Any other comments regarding the weather st ation.	TEXT other_comments_ws

APPENDIX A — CATEGORIES

[1] Categories_LikertScale
Categories: 1: Strongly agree , 2: Agree , 3: Neither agree nor disagree , 4: Disagree , 5: Strongly disagree

[2] Categories_activity_time
Categories: 1: Sunrise (5:00-8:00am) , 2: Morning (8:00am-12:00)noon , 3: Midday (12:00noon-14:00) , 4: Afternoon (14:00-17:30) , 5: Evening (17:30-20:00) , 6: Night (20:00-5:00am)

[3] Categories_Garmin
Categories: 1: GL-3104019 , 2: GL-3104020 , 3: GL-3104021 , 4: GL-3104022 , 5: GL-3104023 , 6: GL-3104024 , 7: GL-3104025 , 8: GL-3104026 , 9: GL-3104027 , 10: GL-3104028 , 11: GL-3104029 , 12: GL-3104030 , 13: GL-3104031 , 14: GL-3104032 , 15: GL-3104033 , 16: GL-3104034 , 17: GL-3104035 , 18: GL-3104036 , 19: GL-3104037 , 20: GL-3104038 , 21: GL-3104039 , 22: GL-3104040 , 23: GL-3104041 , 24: GL-3104042 , 25: GL-3104043 , 26: GL-3104044 , 27: GL-3104045 , 28: GL-3104046 , 29: GL-3104047 , 30: GL-3104048 , 31: GL-3104049 , 32: GL-3104050 , 33: GL-3104051 , 34: GL-3104052 , 35: GL-3104053 , 36: GL-3104054 , 37: GL-3104055 , 38: GL-3104056 , 39: GL-3104057 , 40: GL-3104058 , 41: GL-3104059 , 42: GL-3104060 , 43: GL-3104061 , 44: GL-3104062 , 45: GL-3104063 , 46: GL-3104064 , 47: GL-3104065 , 48: GL-3104066 , 49: GL-3104067 , 50: GL-3104068 , 51: GL-3104069 , 52: GL-3104070 , 53: GL-3104071 , 54: GL-3104072 , 55: GL-3104073 , 56: GL-3104074 , 57: GL-3104075 , 58: GL-3104076 , 59: GL-3104077 , 60: GL-3104078 , 61: GL-3104079 , 62: GL-3104080 , 63: GL-3104081 , 64: GL-3104082 , 65: GL-3104083 , 66: GL-3104084 , 67: GL-3104085 , 68: GL-3104086 , 69 : GL-3104087 , 70: GL-3104088 , 71: GL-3104089 , 72: GL-3104090 , 73: GL-3104091 , 74: GL-3104092 , 75: GL-3104093 , 76: GL-3104094 , 77: GL-3104095 , 78: GL-3104096 , 79: GL-3104097 , 80: GL-3104098 , 81: GL-3104099 , 82: GL-3104100 , 83: GL-3104101 , 84: GL-3104102 , 85: GL-3104103 , 86: GL-3104104 , 87: GL-3104105 , 88: GL-3104106 , 89: GL-3104107 , 90: GL-3104108 , 91: GL-3104109 , 92: GL-3104110 , 93: GL-3104111 , 94: GL-3104112 , 95: GL-3104113 , 96: GL-3104114 , 97: GL-3104115 , 98: GL-3104116 , 99: GL-3104117 , 100: GL-3104118

[4] Categories_Sensors
Categories: 1: Garmin VivoSmart 5 (black wristband) , 2: None

[5] Categories_Activity_Diary
Categories: 1: working on the farm/field/harvesting , 2: cooking , 3: animal husbandry , 4: taking care of children , 5: taking care of family members , 6: getting water from the well , 7: going to school , 8: doing sport , 9: selling products at the market , 10: buying , 11: I rested , 12: watching TV , 13: going to a bar/restaurant , 14: working in a sitting position inside/office work , 15: working in a sitting position outside , 16: working by hand/manual/casual work , 17: driving , 18: bicycling , 19: walking

[6] Categories_Yes_No_Asthma
Categories: 2: Yes , 0: No

[7] Categories_Asthma
Categories: 1: Cough , 2: Chest tightness , 3: Wheeze , 4: Shortness of breath

LEGEND

Legend and structure of information in this file

Name of section

Enabling condition for this section

Question title

Type of question, scope

Variable name

Answer options

SECTION 5: OTHER INCOME SOURCES

E s4_other_sources_which.Contains(98)

Duis aute irure dolor in reprehenderit in voluptate velit esse cillum dolore eu fugiat nulla pariatur?

MULTI-SELECT
SCOPE: PREFILLED

s4_rel_leaders_other

I This refers to family relations
E s3_time_other > 0
V1 s4_rel_leaders_which.Contains(98)
M1 Can not be itself
V2 (s3_time_other_breeding_advice <= (50 - s3_time_art_insem_advice)) ||s3_time_other_breeding_advice == 0
M2 This person is not in the list
F optioncode != s5_ignored_option_code

01 ☐ Community animal health workers
02 ☐ Private
03 ☐ Government
04 ☐ Livestock keepers association
05 ☐ NGO

And 5 other [13]

Additional information:
"I" — Question instruction
"E" — Enabling condition
"V1" — Validation condition №1
"M1" — Message for validation №1
"F" — Filter in Categorical questions

Link to full set in appendix

Breadcrumbs

Type or roster

Roster Title

CHAPTER 3 IDENTIFICATION /
Roster: LEADER RELATION DETAILS
generated by fixed list:

01 Ward Livestock Officer
02 Village Livestock Officer
99 Other (specify)

List items
