# Supplementary material for: Developing Climate Change and Health Impact Monitoring with eHealth at the South East Asia Community Observatory and Health and Demographic Surveillance Site, Malaysia (CHIMES)
Source: Front Public Health. 2023 Dec 1;11:1153149. doi: 10.3389/fpubh.2023.1153149 (PMC10732307; doi:10.3389/fpubh.2023.1153149)
Supplement: Supplementary file 2 [file Data_Sheet_2.PDF]

Informed Consent Form

For participating in the following study:

CHIMES
(**C**limate change and **H**ealth **I**mpact **M**onitoring through **e**Health at **S**EACO HDSS)

Hereby, I

First Name	Last Name	Date of Birth	Participant code

Declare that: ………………………………………………………………

has informed me in oral and written form on the nature, meaning and risks of the scientific investigation within the above-mentioned study, and that I had ample opportunity to clarify my questions with the study team member.

I have bindingly been assured that I can cancel my consent at any time without giving reasons and without any detrimental consequences, that I can object the further analysis of my data and samples and that I can demand their elimination. I have received a copy of the written Participant Information (General Participants) and of the Informed Consent Form (version 0.1, dated 18.01.2023).

I declare, that I voluntarily agree to participate in this scientific study.
I agree that:

1. My personal data which are necessary for the purpose of the above-mentioned study are collected by the above-named study team member on behalf of the principal investigators, Prof. Dr. Tin Tin Su, Director SEACO, Monash University and Dr. sc. hum. Sandra Barteit, Heidelberg Institute of Global Health (HIGH), Universitaetsklinikum Heidelberg, Im Neuenheimer Feld 130.3, 69120 Heidelberg, and are stored and processed, including on electronic data media;

2. That the study results are published in anonymous form which does not allow any conclusions on my person;

3. . **and that for the purpose of the above-mentioned study my data are made available to members of the project (CHIMES).**

Addresses of involved institutions:

a) SEACO (South East Asian Community Observatory), Monash University, 125 Jalan Sia Her Yam, Kampung Abdullah, 85000 Segamat, Johor, Malaysia

b) Heidelberg Institute of Global Health (HIGH), Universitaetsklinikum Heidelberg, Im Neuenheimer Feld 130.3, 69120 Heidelberg, Germany

....

Place, Date Signature of participant

Statement of person witnessing consent (Process for Non-Literate Participants):

……………………………………………. (Name of Witness)

certify that information given to

.... (Name of Participant),

in the local language, is a true reflection of what I have read from the study Participant Information (General Participants), attached.

WITNESS' SIGNATURE (maintain if participant is non-literate)

……………………………………….

Hereby, I declare that I have informed in oral and written form the participant on the date of..........................about nature, meaning, and risks of the above-mentioned study, that I have answered all questions, and that I have handed over copies of the Participant Information and the Informed Consent Form over to the study participant.

....……..

Place, Date Name and signature of informing study team member

Data protection

As mentioned, sensitive personal data will be collected as part of the study.
We are obliged to protect this data and to obtain your consent before the study begins with respect to the DSGVO.
Please read the following text on data protection carefully.

I have been informed and voluntarily agree that my data collected in the study, in particular information about my health, may be recorded in pseudonymized form for the purposes described in the information leaflet, evaluated and possibly also passed on in pseudonymized form to researchers, possibly also in countries with lower data protection requirements than in the European Union. Third parties do not gain access to personal records. Possible recipients of the data include universities and other recognized research institutions that have a legitimate interest in using the data for research purposes. My name will also not be mentioned when the results of the study are published. The personal data will be anonymized as soon as this is possible according to the research purpose. The data will be kept for a maximum of 10 years after completion of the study. I am aware that this consent can be revoked at any time in writing or verbally without giving reasons and without any disadvantage to me. The lawfulness of the data processing carried out until the revocation is not affected by this. In this case, I can decide whether the data collected from me should be deleted or may continue to be used for the purposes of the study.

....
Place, Date Signature of participant
